# Inflammatory Response and Oxidative Stress as Mechanism of Reducing Hyperuricemia of *Gardenia jasminoides*-*Poria cocos* with Network Pharmacology

**DOI:** 10.1155/2021/8031319

**Published:** 2021-12-07

**Authors:** Lijun Liu, Shengjun Jiang, Xuqiang Liu, Qi Tang, Yan Chen, Jiaojiao Qu, Li Wang, Qiang Wang, Yanli Wang, Jinmei Wang, Yan Zhang, Wenyi Kang

**Affiliations:** ^1^Huaihe Hospital, Henan University, Kaifeng 475004, China; ^2^National R&D Center for Edible Fungus Processing Technology, Henan University, Kaifeng, 475004 Henan, China; ^3^Joint International Research Laboratory of Food & Medicine Resource Function, Henan, Kaifeng 475004, China; ^4^Functional Food Engineering Technology Research Center, Henan, Kaifeng 475004, China; ^5^National Health Commission Key Laboratory of Birth Defect Prevention, Henan Institute of Reproductive Health Science and Technology, Zhengzhou 450002, China; ^6^Hebei Food Inspection and Research Institute, Shijiazhuang 050091, China

## Abstract

Hyperuricemia (HUA) is a metabolic disease, closely related to oxidative stress and inflammatory responses, caused by reduced excretion or increased production of uric acid. However, the existing therapeutic drugs have many side effects. It is imperative to find a drug or an alternative medicine to effectively control HUA. It was reported that *Gardenia jasminoides* and *Poria cocos* could reduce the level of uric acid in hyperuricemic rats through the inhibition of xanthine oxidase (XOD) activity. But there were few studies on its mechanism. Therefore, the effective ingredients in *G. jasminoides* and *P. cocoa* extracts (GPE), the active target sites, and the further potential mechanisms were studied by LC-/MS/MS, molecular docking, and network pharmacology, combined with the validation of animal experiments. These results proved that GPE could significantly improve HUA induced by potassium oxazine with the characteristics of multicomponent, multitarget, and multichannel overall regulation. In general, GPE could reduce the level of uric acid and alleviate liver and kidney injury caused by inflammatory response and oxidative stress. The mechanism might be related to the TNF-*α* and IL-7 signaling pathway.

## 1. Introduction

Hyperuricemia (HUA) is the biochemical basis of gout, mainly caused by the decreased excretion or increased production of uric acid or both. In recent years, the number of patients with HUA has increased significantly, and it has shown a development trend of rejuvenation [1]. Uric acid is produced by xanthine oxidase (XOD), which could promote the activation of reduced coenzyme II (NAPDH) and the release of reactive oxygen species (ROS), causing renal oxidative stress [2, 3]. Uric acid at normal concentrations exerts antioxidant effects *in vivo* but above normal physiological levels can act as a prooxidant to expand the body's oxidative stress damage [4–6]. While oxidative stress responses can activate inflammatory factors in kidney cells and induce innate immune responses *in vivo*, the activation of related proinflammatory factors induces the development of inflammation [7–9]. Hyperuricemia has been reported to be closely related to oxidative stress and inflammatory responses [10]. At present, the drugs for the treatment of HUA in clinical practice including allopurinol, benzbromarone, and sodium bicarbonate can control the level of uric acid, but they have a series of toxic side effects such as liver and kidney function injury and hematopoietic dysfunction [11, 12]. Therefore, it is a hotspot to find the low-toxic and efficient antigout drugs from traditional Chinese medicine.

The fruit of *Gardenia jasminoides* is a traditional Chinese medicine which is commonly used to cure fever, red swelling, and pain [13]. *Poria cocos* could promote diuresis and dampness, strengthen the spleen, and calm the heart and is mainly used for the treatment of edema and diuretic detumescence medicine [14, 15]. *G. jasminoides* extracts can reduce uric acid levels in hyperuricemic mice by inhibiting XOD activity [16], but there are few studies on its mechanism.

Network pharmacology is a new method that integrates chemoinformatics, bioinformatics, traditional pharmacology, biological networks, and network analysis. Based on network pharmacology, the comprehensive network construction of medicinal components-active targets-disease targets of Chinese medicine can make the generalization of the pharmacological characteristics of Chinese medicine more comprehensive. In this study, the network pharmacology method combined with molecular docking was used, and the active ingredients in *G. jasminoides* and *P. cocoa* extracts (GPE) were used as the research objects to explore the efficacious ingredients, active targets, and potential mechanisms of GPE to reduce the high uric acid. Furthermore, animal experiments were carried out to verify the pharmacological effects of GPE.

## 2. Materials and Methods

### 2.1. Materials and Reagents

#### 2.1.1. Instrument

Ultraperformance liquid chromatograph was purchased from Waters (Massachusetts, USA). High-resolution mass spectrometer (Q Exactive) was purchased from Thermo Fisher Scientific (Massachusetts, USA). Hypersil GOLD aQ column (100 mm × 2.1 mm, 1.9 *μ*m) was purchased from Thermo Fisher Scientific (Massachusetts, USA). A low-temperature high-speed centrifuge (Centrifuge 5430) was purchased from Eppendorf (Hamburg, Germany). A vortex finder (QL-901) was purchased from Qilinbeier Instrument Manufacturing Co., Ltd. (Haimen, China). A pure water meter (Milli-Q) was purchased from Integral Millipore Corporation (Massachusetts, USA).

#### 2.1.2. Reagent

d_3_-Leucine, ^13^C_9_-phenylalanine, d_5_-tryptophan, and ^13^C_3_-progesterone were used as internal standard. Methanol (A454-4) and acetonitrile (A996-4) were both chromatographic reagents, which were purchased from Thermo Fisher Scientific (Massachusetts, USA). Ammonium formate (17843–250 G) was obtained from Honeywell Fluka (New Jersey, USA). Formic acid (50144–50 mL) was obtained from DIMKA (Los Angeles, USA).

#### 2.1.3. Materials

*Gardenia jasminoides* Ellis (1 kg) and *Poria cocos* (Schw.) Wolf (1 kg) were purchased from the Yuzhou Medicinal Material Market (Yuzhou, China) and identified as *Gardenia jasminoides* Ellis (Rubiaceae) and *Poria cocos* (Schw.) by Professor Changqin Li at Henan University (Kaifeng, China). The voucher specimen (20200718) was deposited in the National Research and Development Center of Edible Fungi Processing Technology, Henan University.

#### 2.1.4. Preparation of Sample

The dried fruits of *G. jasminoides* and the dried sclerotia of *P. cocos* (1 : 1) were soaked in 15 L of water and decocted for 60 min and then filtered. The filter residues were decocted in 10 L of water for 30 min and filtered. The two filtrates were combined and freeze-dried to obtain GPE with a yield of 11%.

### 2.2. Chromatographic Methods

#### 2.2.1. Chromatographic Conditions

Hypersil GOLD aQ column (100 mm × 2.1 mm, 1.9 *μ*m) was used. The mobile phase was 0.1% formic acid-water (liquid A) and 0.1% formic acid-acetonitrile (liquid B) with the sequence as 0–2 min 5% B; 2–22 min 5–95% B; 22–27 min 95% B; 27.1–30 min 5% B. The flow rate was 0.3 mL/min, the column temperature was 40°C, and the injection volume was 5 *μ*L [17].

#### 2.2.2. Mass Spectrometry Conditions

The mass range was set at 150–1500 daltons, the MS resolution was 70000, the AGC was 1*e*^6^, and the maximum injection time was 100 ms. According to the strength of the MS ions, the top 3 peaks were selected for fragmentation. The MS^2^ resolution was 35000, AGC is 2*e*^5^, the maximum injection time was 50 ms, and the fragmentation energy was set as 20, 40, and 60 eV. Ion source (ESI) parameter settings are as follows: sheath gas flow rate was 40 arb, aux gas flow rate was 10 arb, spray voltage of positive ion mode was 3.80 kV, spray voltage of negative ion mode was 3.20 kV, ion capillary temp was 320°C, and aux gas heater temp was 350°C [17].

#### 2.2.3. Data Analysis

UPLC-MS/MS technology was used to systematically analyze the chemical constituents of GPE with positive and negative ion modes, respectively. The compounds were identified by comparing the retention time, accurate molecular weight, and MS^2^ data with standard databases such as MZVault, MZCloud, and BGI Library (self-built standard Library by BGI Co., Ltd.).

### 2.3. Screening and Target Prediction of Active Compounds in GPE

The effective ingredients of GPE were screened by TCMSP database and LC-MS-MS identification results. The screening condition is oral bioavailability (OB) ≥ 30%. Drug likeness (DL) is greater than ≥0.18. At the same time, the target prediction of the Swiss target prediction platform is used. UniProt is used to standardize the names of the screened target proteins.

### 2.4. Acquisition of HUA Disease Targets

“High uric acid” as a keyword was used to search and screen databases such as GeneCards, Online Mendelian Inheritance in Man (OMIM), PharmGkb, TTD, and DrugBank. Venny 2.1.0 was used to draw a Venn diagram to obtain HUA disease target.

### 2.5. Construction and Analysis of Protein Interaction Network

Venny 2.1.0 was used to draw a Venn diagram and to obtain the intersection of GPE and the effective ingredients with the hyperuricemia target, which was the possible target of GPE in the treatment of hyperuricemia, and the intersection would be the target. A PPI protein interaction network was established in the database of the biomolecular functional annotation system STRING (https://string-db.org/), and the analysis was performed based on the results.

### 2.6. Topological Analysis of Protein Interaction Network (CytoNCA)

The PPI protein interaction files was imported and obtained from the STRING (https://string-db.org/) database into Bisogenet of Cytoscape to construct the PPI protein interaction network. CytoNCA in Cytoscape was used to perform topological analysis on the interaction network. The core network and key proteins for the treatment of HUA were obtained.

### 2.7. GO and KEGG Enrichment Analysis

Gene Ontology (GO) was used to mainly analyze the gene and protein functions of various species from the three aspects: biological process (BP), cellular component (CC), and molecular function (MF). Kyoto Encyclopedia of Genes and Genomes (KEGG) pathway enrichment analysis is commonly used to clarify the role of target proteins in signaling pathways. In this study, the clusterProfiler program package in R language was used to perform GO enrichment analysis and KEGG (Kyoto Encyclopedia of Genes and Genomes) pathway analysis on shared targets. The corresponding target proteins in the GPE were directly mapped on the pathway where the drug target is enriched as the pathway for drug therapy, and a bubble chart is drawn for visualization.

### 2.8. Molecular Docking

The 3D structure of the compound in GPE was obtained from PubChem (https://pubchem.ncbi.nlm.nih.gov/) and screened from the RCSB PDB (http://www.rcsb.org/) database. The water molecules and small ligand molecules of the core target protein from the crystal structure were removed, and the structure of the potential protein isolated. The genetic algorithm in the SYBYL-X software was used for semiflexible docking. The center coordinates and size of the box were set according to the position of the active site of the protein molecule and the area where it might have an effect on the small molecule of the ligand. The remaining parameters remained as default. The target protein was subjected to molecular docking analysis.

### 2.9. Animal Experiments

#### 2.9.1. Materials and Reagents

Potassium oxonate was purchased from Yuanye Biological Co., Ltd. (Shanghai, China). Benzbromarone was purchased from Heumann Pharma GmbH (Kunshan, China). CMC-Na was purchased from Shanghai Chemical Reagent Station Branch Factory (Shanghai, China). Uric acid (UA) content detection kit and XOD activity detection kit were purchased from Solaibao Biotechnology Co., Ltd. (Beijing, China). Total superoxide dismutase (SOD), catalase (CAT), malondialdehyde (MDA), glutathione peroxidase (GSH-Px), total antioxidant capacity (T-AOC), blood urea nitrogen (BUN), creatinine (Cr), interleukin-1*β* (IL-1*β*), interleukin-6 (IL-6), interleukin (IL-2), interleukin-4 (IL-4), and tumor necrosis factor-*α* (TNF-*α*) detection kits were purchased from Nanjing Jiancheng Biotechnology Co., Ltd. (Nanjing, China). NADPH oxidase (NADPH-OX) kit, reactive oxygen species (ROS) kit, and *β*2 microglobulin (*β*2-MG) kit were purchased from Shanghai Jining Biotechnology Co., Ltd. (Shanghai, China).

#### 2.9.2. Animals and Experimental Design

*(1) Animals*. Forty Specific Pathogen-Free (SPF) Sprague-Dawley (SD) male rats were provided by the Henan Laboratory Animal Center (laboratory animal license number: SCXK (Yu) 2017-0001). The rats were adapted for one week (temperature 25 ± 2°C, light circle 12 h/d, humidity 40 to 45%) before the experiment and fed with a standard diet with free access to water.

#### 2.9.3. Experimental Grouping and Administration

Thirty-six rats were randomly divided into six groups: blank control (BC) group, model control (MC) group, positive control (PC) group, GPE high-dose group (GPE-HD, 1000 mg/kg), medium-dose group (GPE-MD, 500 mg/kg), and low-dose group (GPE-LD, 250 mg/kg). The dosage of benzbromarone for the PC group was 8 mg/kg, and it was suspended with an appropriate amount of 0.5% CMC-Na. The BC group was administered with the same volume of 0.5% CMC-Na without benzbromarone.

#### 2.9.4. Establishment of Hyperuricemic Rat Model

Animal groups of GPE-HD, GPE-MD, and GPE-LD were administered for 15 d. Except for the blank group, the other groups were intraperitoneally injected with 300 mg/kg potassium oxonate 1 h before the last oral administration, and the blank group was injected with an equal volume of 0.5% CMC-Na. Two hours after the last administration, blood was collected from the abdominal aorta and centrifuged. The supernatant was collected by centrifugation at 3500 g for 10 min. The tissues of the liver and kidney were collected and frozen in a -80°C freezer.

#### 2.9.5. Determination of Inflammatory Factors in Rat Plasma

The contents of IL-2, IL-6, TNF-*α*, IL-4, and IL-1*β* in plasma were measured according to the ELISA kit instructions.

#### 2.9.6. Determination of Antioxidative Stress Ability in Rats

The liver tissues were collected to make tissue homogenates at the corresponding concentrations, and the activities of XOD, SOD, NADPH-OX, ROS, GSH-PX, T-AOC, CAT, and MDA in plasma and tissues were measured, respectively, according to the kit instructions.

#### 2.9.7. Effects of Samples on Liver and Kidney Function Injury

The contents of BUN, Cr, *β*_2_-MG, and XOD in plasma were determined in strict accordance with the kit instructions.

#### 2.9.8. Pathological Changes of Liver and Kidney Tissues

Two hours after the last oral administration, the liver and kidney tissues were collected, washed with cold saline, fixed in 4% (wt/vol) paraformaldehyde solution for 24 h, rinsed in PBS for 6 h, dehydrated and embedded, then sliced into 5 *μ*m sections by a microtome, stained with H&E, and observed and photographed under a microscope.

#### 2.9.9. Detection of Uric Acid Levels in Rat Plasma

The content of UA in plasma was measured within 15-20 min in strict accordance with the kit instructions.

#### 2.9.10. Statistical Processing

SPSS 19.0 software was used to process statistical data. Differences of data were analyzed by one-way ANOVA. Results were expressed as mean ± standard deviation (SD). *P* < 0.05 was considered significantly different.

## 3. Results and Analysis

### 3.1. Effective Ingredient Screening and Target Prediction in GPE

Based on TCMSP database and LC-MS-MS identification results, the effective ingredients and target prediction data of GPE were selected (in Tables 1 and 2), and 30 effective compounds and corresponding gene targets of the GPE were obtained. The specific effective ingredients from GPE are illustrated in Table 3.

### 3.2. Intersection Analysis of the Effective Ingredient Targets of GPE and the Targets of HUA

**Figure 1:**
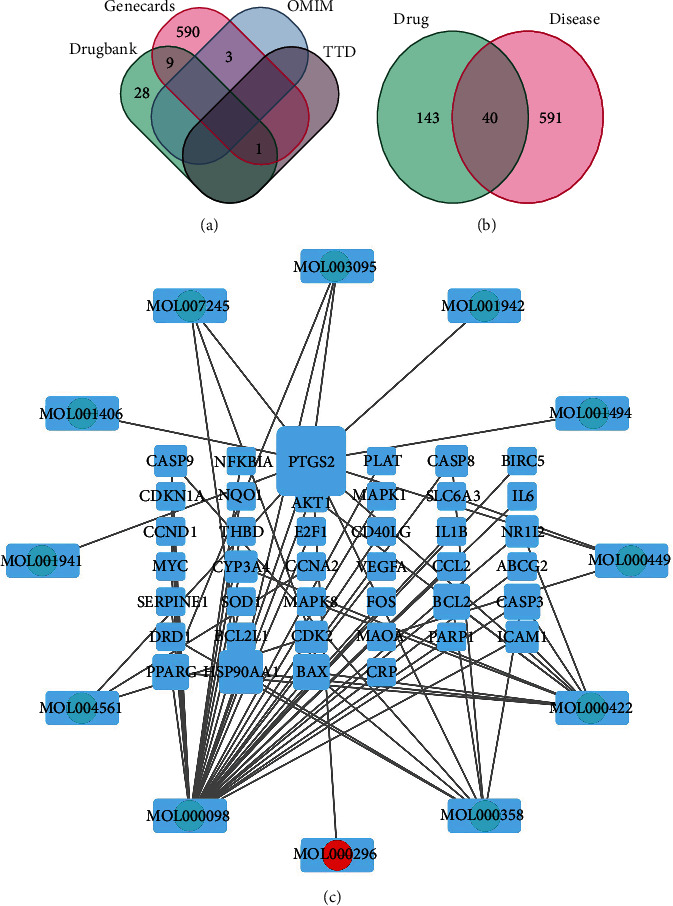
(a) HUA disease targets; (b) intersection of active component targets of GPE and hyperuricemic acid target; (c) regulation network of active components of GPE.

The 631 hyperuricemia-related targets were searched (Figure 1(a)) from GeneCards, Online Mendelian Inheritance in Man (OMIM), PharmGkb, TTD, DrugBank, and other databases. There were 183 targets of GPE intersected with the disease targets, and 40 common targets of GPE and HUA were mapped as the Venn diagram (in Figure 1(b)).

### 3.3. GPE Effective Ingredient-Disease Target Regulatory Network

Twelve effective ingredients and 40 related targets of GPE were introduced into Cytoscape 3.7.0 to obtain the “effective ingredient-target” regulatory network diagram (Figure 1(c)). In Figure 1(c), there were 52 nodes (12 ingredients, 40 targets) and 70 edges; round nodes represent active ingredients, square nodes represent corresponding targets, and black edges represent the relationship between active ingredients and HUA targets. The results showed that 58.3% (7) of the effective ingredients in GPE could act on multiple gene targets. Among them, quercetin, kaempferol, *β*-sitosterol, and hederagenin had many targets and played the important role in the regulation process. The same target could also be regulated by multiple effective ingredients. Thirteen targets (32.5%) were corresponding to more than two effective ingredients, indicating the complex and diverse action mechanism characteristics of GPE components.

### 3.4. Results of Core Target Screening and PPI Network Construction

**Figure 2:**
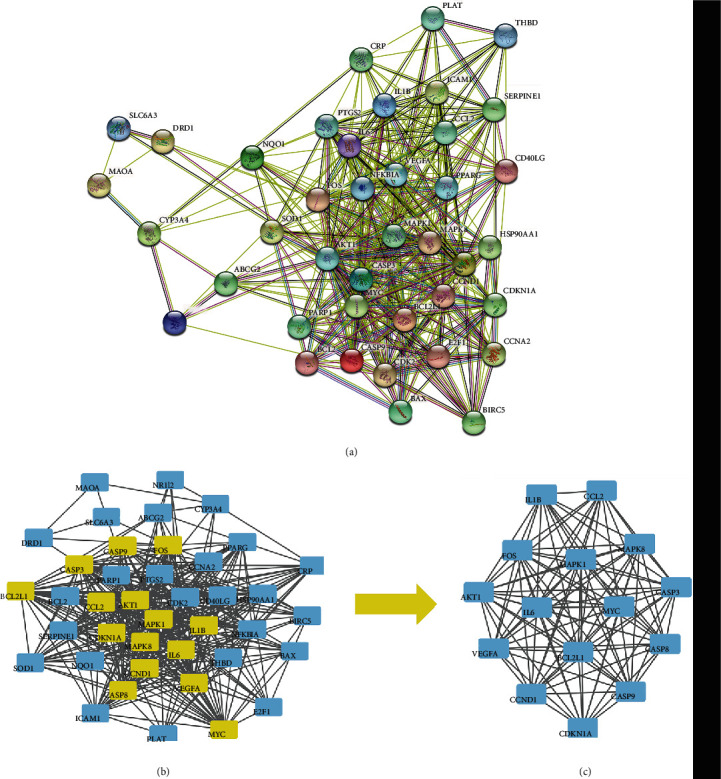
PPI protein interaction network of GPE in treatment of HUA targets (a) and CytoNCA Core Network Analysis (b, c).

The intersection target was imported into the STRING database, and the species was set as “Homo sapiens” to analyze protein interaction (Figure 2(a)). The relevant files were imported into the Cytoscape software to build a PPI network diagram; one node represented one target, and the TCMSP edge represented the target. We click on the interaction relationship and finally get the protein interaction network analysis diagram (Figure 2(a)). The network contained 15 key gene targets, i.e., IL-1B, IL-6, MAPK1, MAPK8, AKT1, MYC, VEGFA, CASP3, CASP8, CASP9, BCL2L1, FOS, CCND1, CDKN1A, and CCL2, which were the components of the core network (Figures 2(b) and 2(c)).

### 3.5. The Results of GO Function Enrichment Analysis

**Figure 3:**
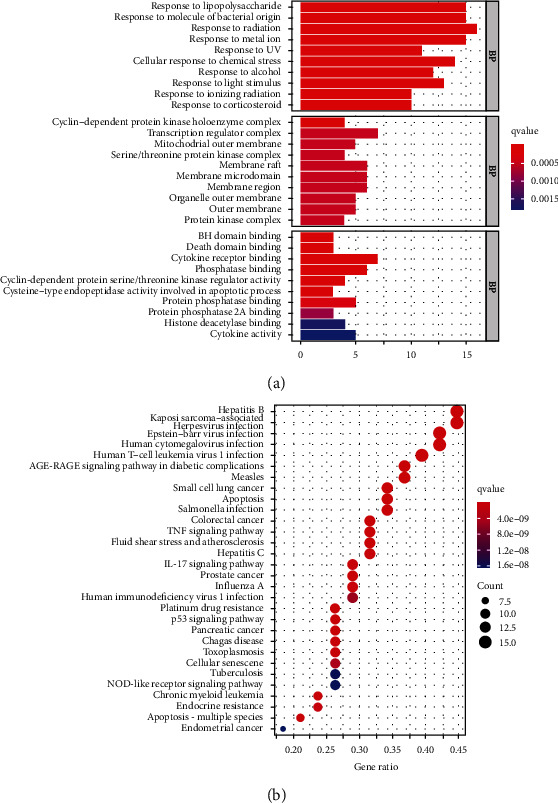
GO biological function enrichment bar chart (a) and KEGG pathway enrichment analysis bubble diagram (b).

GO describes the biological process (BP) of gene products (protein or RNA), molecular function (MF), and cellular component (CC) and organizes the functional concepts with different thicknesses into atlases for analysis and sorting according to *P* value (Figure 3(a)). In Figure 3(a), the abscissa represents the number of enriched genes and the color of the bar represents the size of the *P* value. When the color changed from blue to red, the *P* value changed to small. The process was mainly concentrated in the biological process, and there were 1591 enrichment results which were mainly involved in the reaction of oxidative stress, lipids, bacteria-derived molecules, and chemical substances. In molecular functions, there were 57 results which were related to cell death, cytokine receptors, and other enzyme activities. There were 27 enriched results for cell composition, which had the relationship with the cyclin-dependent protein kinase holoenzyme complex, transcription regulation complex, and mitochondrial outer membrane.

### 3.6. The Results of KEGG Pathway Enrichment Analysis

The corresponding target protein of GPE was mapped to the pathway by KEGG to analyze the activity, target, and pathway of drug. The signaling pathways mainly included the inflammatory signaling pathways of TNF and IL-7, apoptosis, tumors such as small cell lung cancer, and AGE-RAGE signaling pathway in diabetic complications. There were 139 pathways by the KEGG enrichment pathway analysis, and the top 20 pathways are shown in Figure 3(b).

### 3.7. Docking Prediction of Effective Ingredients and Core Target Molecules

**Figure 4:**
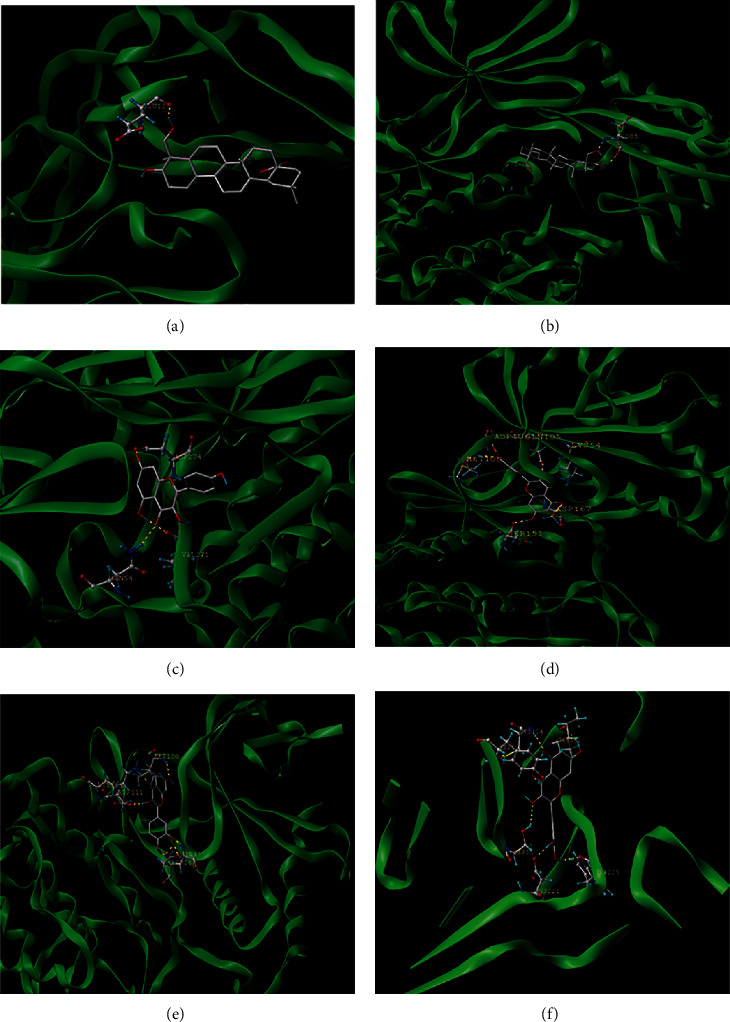
Molecular binding map of effective ingredients in GPE with HUA potential target: (a) hederagenin AKT1; (b) hederagenin VEGFA; (c) kaempferol AKT1; (d) kaempferol MAPK1; (e) quercetin IL-6; (f) quercetin MAPK1.

The core targets of IL-6, AKT1, MYC, VEGFA, and MAPK1 protein with higher node degree values and the best effective ingredients, quercetin, kaempferol, and hederagenin, in GPE were selected for molecular docking in the PPI protein interaction network (Table 4, Figure 4).

### 3.8. Verification of Animal Experiment

#### 3.8.1. Effects on Plasma Uric Acid Levels of Hyperuricemic Rats

**Figure 5:**
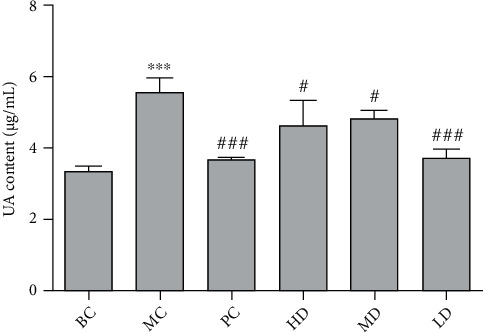
Effect of GPE on UA levels in hyperuricemic rats. Compared with BC, ^∗^*P* < 0.05, ^∗∗^*P* < 0.01, ^∗∗∗^*P* < 0.001; compared with MC, ^#^*P* < 0.05, ^##^*P* < 0.01, ^###^*P* < 0.001.

In Table 5 and Figure 5, the levels of uric acid in the model group were significantly increased compared with the blank group (*P* < 0.001), which indicated that the hyperuricemic rat model was established. Compared with the MC, GPE-HD, GPE-MD, and GPE-LD could effectively reduce the levels of uric acid (*P* < 0.05). GPE-LD showed significant difference (*P* < 0.001), and there was no significant difference compared with the PC (*P* > 0.05).

#### 3.8.2. Effect on Antioxidative Stress Ability of Hyperuricemic Rats

**Figure 6:**
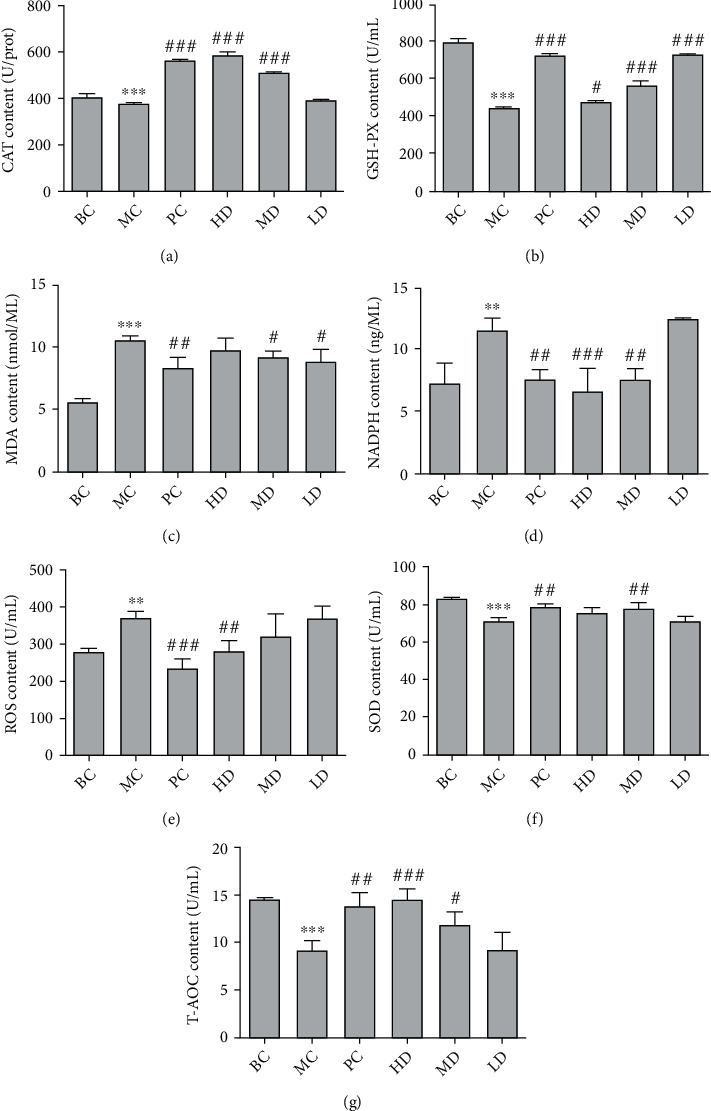
Effect of GPE on the levels of CAT (a), GSH-PX (b), MDA (c), NADPH (d), ROS (e), SOD (f), and T-AOC (g) in hyperuricemic rats. Compared with BC, ^∗^*P* < 0.05, ^∗∗^*P* < 0.01, ^∗∗∗^*P* < 0.001; compared with MC, #*P* < 0.05, *^##^P* < 0.01, *^###^P* < 0.001.

In Figures 6(b), 6(f), and 6(g), compared with BC, the contents of GSH-PX, T-AOC, and SOD in the plasma of MC were significantly decreased (*P* < 0.001), and the contents of CAT in the liver homogenate were significantly decreased (*P* < 0.001). In Figures 6(a), 6(c), 6(d), and 6(e), compared with BC, the contents of ROS, MDA, and NADPH in the plasma of MC were significantly increased (*P* < 0.01, *P* < 0.001, and *P* < 0.01, respectively); compared with MC, the levels of GSH-PX, T-AOC, and SOD in plasma of GPE-HD, GPE-MD, and GPE-LD were significantly increased (*P* < 0.05), and the contents of CAT in the liver homogenate of rats were significantly increased (*P* < 0.05). The contents of ROS, MDA, and NADPH in the plasma of GPE-HD, GPE-MD, and GPE-LD were significantly decreased (*P* < 0.05), indicating that GPE could improve oxidative stress produced by hyperuricemia.

#### 3.8.3. Effect on Liver and Kidney Function in Hyperuricemic Rats

**Figure 7:**
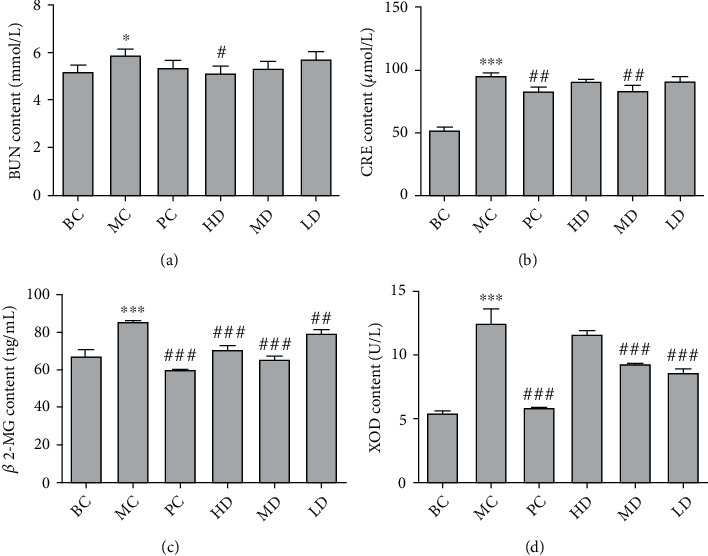
Effects of GPE on BUN (a), CRE (b), *β2*-MG (c), and XOD (d) levels in hyperuricemic rats. Compared with BC, ^∗^*P* < 0.05, ^∗∗^*P* < 0.01, ^∗∗∗^*P* < 0.001; compared with MC, ^#^*P* < 0.05, ^##^*P* < 0.01, ^###^*P* < 0.001.

In Figure 7, compared with the BC, the contents of BUN, CRE, *β*2-MG, and XOD in the MC were significantly increased (*P* < 0.05); compared with the MC, the GPE-HD, GPE-MD, and GPE-LD could significantly reduce the BUN, CRE, *β*2-MG, and XOD in the plasma of rats (*P* < 0.05). Among them, the contents of *β*2-MG in the plasma of rats in the GPE-MD and GPE-HD were extremely significantly decreased (*P* < 0.001). It indicated that GPE could reduce uric acid in the plasma of hyperuricemic rats by reducing XOD activity and could improve liver and kidney damage produced by HUA.

#### 3.8.4. Determination of Inflammatory Factors in Rat Plasma

**Figure 8:**
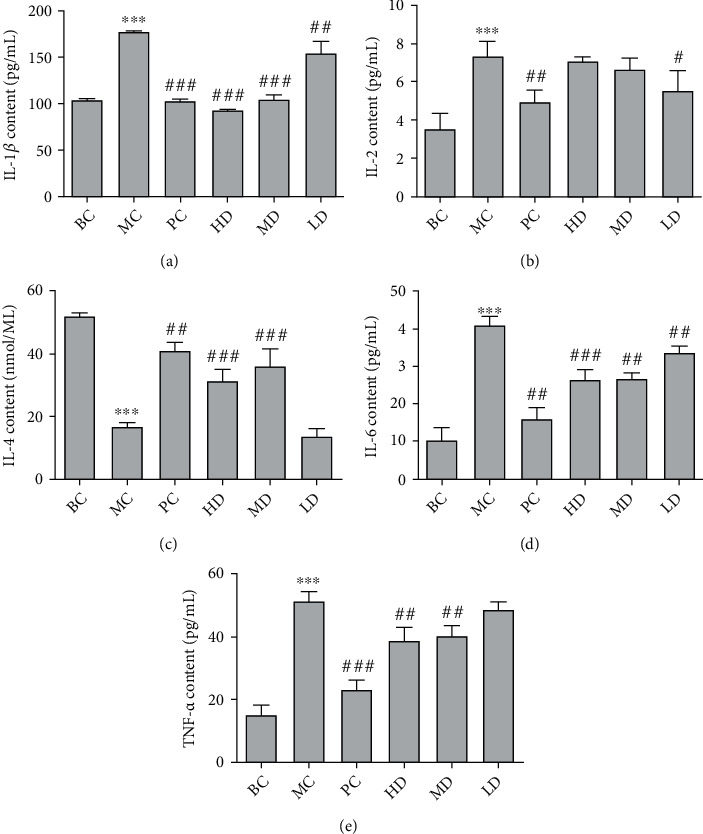
Effect of GPE on the contents of IL-1*β* (a), IL-2 (b), IL-4 (c), IL-6 (d), and TNF-*α* (e) levels in hyperuricemic rats. Compared with BC, ^∗^*P* < 0.05, ^∗∗^*P* < 0.01, ^∗∗∗^*P* < 0.001; compared with MC, ^#^*P* < 0.05, ^##^*P* < 0.01, ^###^*P* < 0.001.

In Figure 8, the expression levels of IL-1*β*, IL-2, IL-6, and TNF-*α* in the plasma in the MC were significantly increased compared with the BC (*P* < 0.001). The contents of IL-2, IL-6, and TNF-*α* in the plasma of the GPE-HD, GPE-MD, and GPE-LD were significantly decreased compared with those of the MC (*P* < 0.05), and the contents of IL-1*β* and IL-6 in the GPE-MD and GPE-HD were significantly decreased (*P* < 0.001). The contents of IL-4 in the plasma in the MC were significantly decreased compared with the those in the BC (*P* < 0.001). Compared with the MC, the IL-4 content in the plasma of the GPE-HD, GPE-MD, and GPE-LD was significantly increased (*P* < 0.001). The results showed that the GPE could improve the inflammatory level of hyperuricemia and also had an anti-inflammatory effect.

#### 3.8.5. Pathological Changes of Renal Tissue in Rats

**Figure 9:**
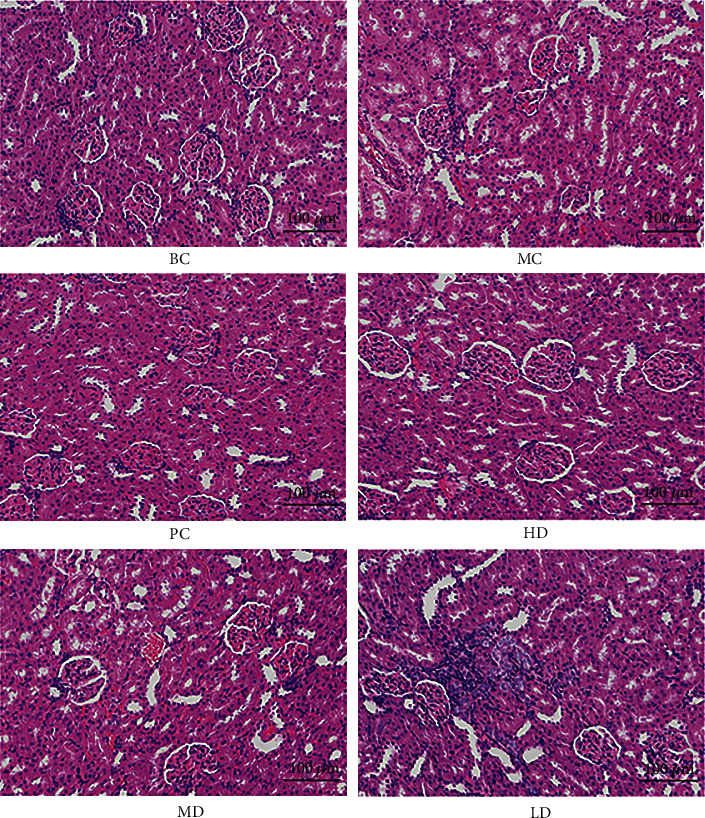
Pathological section of kidney tissue of hyperuricemic rats (200x).

In Figure 9, the renal structure of BC was normal without inflammatory cell infiltration. In the MC group of rats, there were disorganized renal tubules, inflammatory factor infiltration in renal interstitial part, renal cortical edema, massive accumulation of lymphocytes, and inflammatory cell infiltration around glomeruli. The number of lymphocytes was relatively reduced in LD compared with MC, but there was still inflammatory cell infiltration, and tubular arrangement was scattered, which was not significant in GPE-MD compared with MC. The glomerular morphological structure of GPE-HD was basically normal with regular tubular arrangement.

#### 3.8.6. Pathological Changes of Liver Tissue in Rats

**Figure 10:**
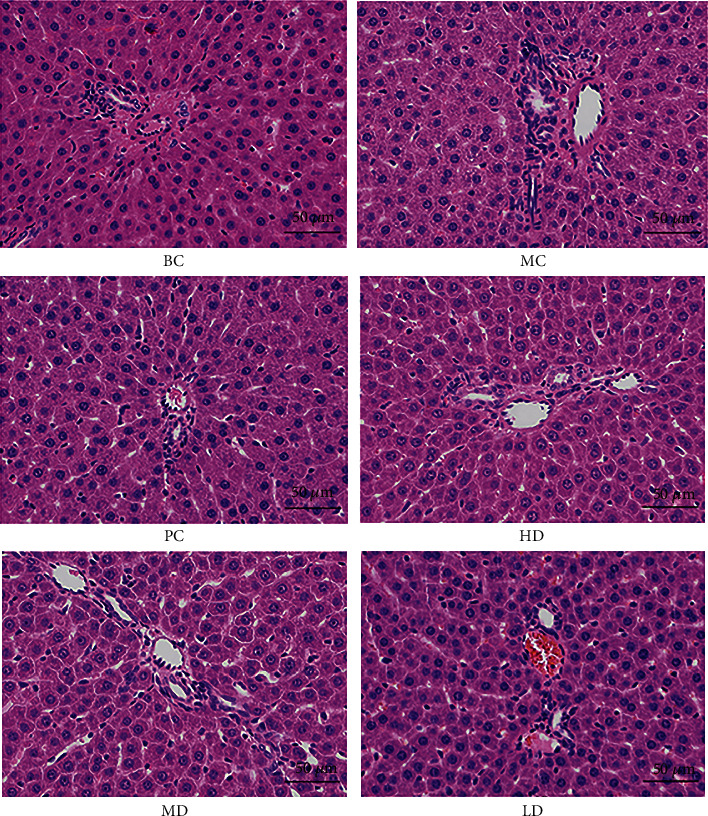
Histopathological section of liver tissue in hyperuricemic rats (400x).

In Figure 10, compared with BC, the hepatocytes of rats in MC were significantly swollen, tiny round fat vacuoles were observed in the cytoplasm, and the swelling of hepatocytes was alleviated to varying degrees in each group without other obvious abnormalities. The swelling of hepatocytes was most significantly alleviated in LD, indicating that GPE could protect the liver from damage.

## 4. Discussion

In recent years, the incidence of gout has increased exponentially. There are two main reasons for excessive uric acid in the body: (i) the excessive production of UA due to abnormal activity of XOD or excessive intake of exogenous purines and (ii) the accumulation of UA in the body due to the decrease of UA excretion [18]. Despite the current drugs that inhibit XOD having a good effect on reducing UA, there are severe adverse reactions such as renal injury and myelosuppression [19]. *Gardenia* has the effect of purging fire and removing annoyance, clearing heat and dampness, and cooling blood and detoxification. Besides, it is often used in diseases such as fever, swelling and pain, astringent pain of gonorrhea, and hematemesis. *Poria cocos* has the effects of promoting diuresis and dampness, invigorating the spleen, and calming the heart [20]. Thus, this study discussed the potential mechanism of GPE in the treatment of HUA based on network pharmacology to understand the pharmacological mechanism of GPE.

Forty core targets of GPE in the treatment of HUA were screened in this study. Further analysis of the compound-disease-target regulatory network showed that quercetin, *β*-sitosterol, kaempferol, hederagenin, and other active ingredients could act on multiple targets in the network. Besides, the same target could also be regulated by multiple effective ingredients. Thirteen targets (32.5%) corresponded to more than two effective ingredients, indicating that there was the complex composition of GPE and the action mechanism of various targets. Quercetin can inhibit monosodium urate- (MSU-) induced mechanical hyperalgesia, leukocyte recruitment, the production of TNF-*α* and IL-1*β*, the production of superoxide anion, the activation of inflammatory reaction, the decrease of antioxidant level, the activation of NF-*κ*B, and the expression of the inflammatory component mRNA [21]. *β*-Sitosterol can improve nephrotoxicity and kidney disease and adjust the activity of NRF-2 antioxidant enzymes to reduce nephrotoxic mouse creatinine and the expression of uric acid, urea, and iNOS to normal levels, and excessive peroxides and internal and external toxicants in the body are eliminated [22]. Hederagenin has antibacterial and anti-inflammatory pharmacological effects, and previous studies have shown that hederagenin can block the NF-*κ*B signaling pathway to reduce the release of inflammatory factors such as IL-6, IFN-*γ*, TNF-*α*, and NO [23]. Some studies have shown that kaempferol may be a potential XOD inhibitor, because it can block the entry of the substrate by inserting the hydrophobic active site of XOD and inhibiting the activity of XOD by competing sites. Moreover, it can reduce the level of serum UA in hyperuricemia rat [24, 25]. PPI network also confirmed that there was a close relationship between GPE and HUA targets, and the results were reliable and of great reference value.

It is preliminarily predicted that GPE plays a role in the treatment of HUA through cell death, cytokine receptor and other enzyme activity pathways, TNF signal pathway, IL-7 signal pathway, and others through the GO functional enrichment analysis and KEGG pathway enrichment analysis of the core targets. During the pathological process of HUA, UA is an important endogenous antioxidant [26]. Under normal circumstances, the ROS generated are neutralized by the endogenous antioxidants, and there is an equilibrium between the ROS generated and the antioxidants present, but the continuous increase of the UA level will enhance the oxidative stress reaction *in vivo* [27]. The increase of the UA level in the blood can destroy the oxidation-reduction balance of the body through various ways, enabling the body to produce a mass of reactive oxygen species (ROS), damaging tissue cells, thus reducing the antioxidant capacity of the body and at last leading to the occurrence of oxidative stress damage [28–30]. NADPH oxidases (Nox) are one of many sources of ROS. Its catalytic product ROS participates in body defense and information transmission. The high level of ROS caused by excessive activation of NADPH will lead to tissue inflammation and further pathological damage of tissues and organs [31]. NADPH oxidase and its ROS products play a key role in the occurrence and development of acute kidney injury [32, 33]. The body scavenges reactive oxygen radicals through active enzymes such as superoxide dismutase (SOD), total antioxidant capacity (T-AOC), and peroxidase GSH-Px, thus reducing cell damage [34, 35]. Studies have shown that TNF-*α* can induce oxidative stress and inflammation. Besides, the increase of ROS production mainly comes from the intracellular NADPH oxidase pathway. TNF-*α* increases the level of oxidative stress mainly by upregulating the expression of NOX2 and its related subunits [36, 37]. IL-7 is a multifunctional cytokine, which can act on a variety of cells. The main function is to promote the development, antiapoptosis, and proliferation of immature B and T cells and to participate in the differentiation and maturation of thymocytes [38, 39]. The biological effect of IL-7 is mainly achieved through the binding of IL-7 to the IL-7 receptor (IL-7R). IL-7 activates the IL-7R signal pathway, upregulates the antiapoptotic protein, inhibits differentiated and activated T cell apoptosis, and downregulates the proapoptotic protein to prevent T cell apoptosis after binding to IL-7R. IL-7 has a strong immunomodulatory effect. As a consequence, IL-7 plays an important role in maintaining the homeostasis of immune function *in vivo* [40, 41].

High uric acid environment can cause oxidative stress, which is closely related to inflammation. Oxidative stress will promote the expression of inflammatory mediators such as TNF-*α* and IL-6 [42]. At the same time, inflammatory cells generate more reactive oxygen species after activation, resulting in an increase of oxidative stress level after inflammatory lesions. Another major cause of the creation of hyperuricemia is the reduction of uric acid excretion. Around 70% of uric acid in the body is excreted by the kidney. Uric acid is finally excreted with urine in the form of urate after glomerular filtration, renal tubular reabsorption, renal tubular secretion, and reabsorption after secretion [43]. *β*2-Microglobulin (*β_2_*-MG) is a small molecule globulin produced by lymphocytes, platelets and polymorphonuclear leukocytes. The excretion of *β_2_*-MG is extremely low under normal conditions, but its excretion increases when renal tubules are damaged or filtration function of glomerular decreases [44, 45]. Blood urea nitrogen (BUN) is one of the indicators of renal function, and it is the final product of human protein metabolism. When glomerular filtration ability decreased to a certain degree, BUN level tends to increase [46]. Serum creatinine (SCr) is an endogenous creatinine filtered by glomerulus. Like BUN, the change of SCr content is also an important indicator of renal function [47]. When renal function is damaged, the level of SCr will increase [48]. In the clinic, the degree of renal function injury of patients is often judged by the detection of SCr index. Some studies have shown that hyperuricemia can damage rats' kidneys through inflammatory reaction and the oxidative stress pathway [49–51].

The model of hyperuricemia rats was established to demonstrate the results of network pharmacological analysis. The results showed that compared with the model group, the GPE group could significantly increase the activities of SOD, CAT, GSH-Px, and T-AOC in the plasma and liver tissue and decrease the levels of ROS, MDA, and NADPH in the plasma. It is suggested that GPE has the effect of antioxidant stress. The secretion of IL-4 was significantly increased after treatment with GPE. However, the contents of IL-6, IL-2, IL-1*β*, and TNF-*α* in the renal tissue of hyperuricemia rats were significantly decreased, indicating that GPE can enhance the secretion of IL-4 in hyperuricemia rats and reduce the inflammatory level of hyperuricemia rats, which has a certain anti-inflammatory effect. Compared with the blank group, the levels of *β*2-MG, BUN, and SCr in the model group were significantly higher, indicating that the kidney of hyperuricemia rats was damaged. The sections of rat renal tissue in the model group showed that the renal tubules were messy, and the renal interstitium was infiltrated with inflammatory factors. It also indicated that the kidney in the model group was damaged, which led to the decline of renal function in hyperuricemia rats. These renal injuries can reduce the renal excretion of UA, thus increasing the level of uric acid in the body. Compared with the model group, the levels of *β*_2_-MG, BUN, and Cr in the GPE group decreased significantly, indicating that the GPE can protect and improve renal function. It is speculated that GPE promotes the excretion of UA by improving the renal function damage caused by oxidative stress, thus reducing the level of UA. Besides, it can inhibit inflammatory reaction and antioxidant stress in order to achieve the purpose of treatment. Generally speaking, GPE has the characteristics of multicomponents, multitargets, and multipathways in the treatment of HUA. It mainly plays a role in controlling the development of HUA through cell death, cytokine receptor, and other enzyme activity pathways, such as TNF *α*, IL-7, and other signaling pathways. Further animal experiments confirmed that GPE can reduce the level of oxidative stress in HUA model rats. In addition, regulating the expression of inflammatory factors such as TNF-*α* in renal tissue may be one of its mechanisms.

## 5. Conclusion

GPE has the characteristics of multicomponents, multitargets, and multipathways in the treatment of HUA. It mainly plays a role in controlling the development of HUA through cell death, cytokine receptor, and other enzyme activity pathways, such as TNF-*α* and IL-7 signaling pathways. Further animal experiments confirmed that GPE can reduce the level of oxidative stress in HUA model rats. In addition, regulating the expression of inflammatory factors such as TNF-*α* in renal tissue may be one of its mechanisms.

## Figures and Tables

**Table 1:** The compounds identified from GPE by LC-MS-MS in positive model.

RT (min)	Adducts	Formula	Measured value	Molecular weight	Name
0.871	[M+H]^+^	C_10_H_14_N_5_O_7_P	347.06293	348.07007	Adenosine 5′-monophosphate
1.012	[M+H]^+^	C_6_H_14_O_6_	182.07906	183.08633	Galactitol
1.115	[M+H]^+^	C_9_H_11_NO_3_	181.07413	182.08142	L-tyrosine
1.12	[M+H]^+^	C_10_H_13_NO_4_	267.09656	268.1037	Adenosine
3.05	[M+H]^+^	C_11_H_9_NO_2_	187.06352	188.0708	Indole-3-acrylic acid
3.834	[M+H]^+^	C_16_H_26_O_8_	346.16224	347.17001	Jasminoside B
3.978	[M+H]^+^	C_10_H_12_N_2_	160.10024	161.10744	Tryptamine
4.18	[M+H]^+^	C_10_H_14_O	150.1046	151.11189	Carvone
4.607	[M+H]^+^	C_14_H_14_N_2_O_5_	290.09014	291.09741	Indole-3-acetyl-l-aspartic acid
5.912	[M+H]^+^	C_15_H_22_O_2_	234.16208	235.16927	Artemisinic acid
6.153	[M+H]^+^	C_33_H_40_O_19_	740.21617	741.22345	Mauritianin
6.216	[M+H]^+^	C_10_H_10_O_4_	194.05802	195.06546	Isoferulic acid
6.274	[M+H]^+^	C_10_H_16_O	152.12026	153.12759	*α*-Pinene-2-oxide
6.295	[M+H]^+^	C_27_H_30_O_16_	610.15294	611.16016	Rutin
6.357	[M+H]^+^	C_27_H_30_O_16_	610.15294	611.15985	Rutin
6.514	[M+H]^+^	C_10_H_12_O_2_	164.08388	165.09126	4-Phenylbutyric acid
6.526	[M+H]^+^	C_21_H_20_O_12_	464.09551	465.10303	Isoquercitrin
6.531	[M+H]^+^	C_15_H_10_O_7_	302.04238	303.04974	Quercetin
6.84	[M+H]^+^	C_27_H_30_O_15_	594.1591	595.16565	Kaempferol-3-*O*-rutinoside
7.252	[M+H]^+^	C_9_H_8_O_3_	164.04749	165.05476	3-Hydroxycinnamic acid
7.407	[M+H-H_2_O]^+^	C_10_H_10_O_4_	194.058	177.05469	Ferulic acid
10.436	[M+H]^+^	C_12_H_17_NO	191.13118	192.13846	DEET
10.839	[M+H]^+^	C_21_H_32_O_2_	316.24007	317.24747	Pregnenolone
11.096	[M+H]^+^	C_15_H_18_O_2_	230.13068	231.13805	Dehydrocostus lactone
12.931	[M+H]^+^	C_24_H_30_O_6_	414.20437	415.21176	Bis(4-ethylbenzylidene)sorbitol
13.036	[M+H]^+^	C_20_H_34_O_2_	306.25575	307.26303	11(*z*),14(*z*),17(*z*)-Eicosatrienoic acid
14.969	[M+H]^+^	C_18_H_30_O_2_	278.22439	279.23169	*Α*-Eleostearic acid
15.833	[M+H]^+^	C_18_H_30_O_3_	294.21933	295.22665	9-Oxo-10(e),12(e)-octadecadienoic acid
16.83	[M+H]^+^	C_22_H_32_O_2_	328.24016	329.24728	Docosahexaenoic acid
17.661	[M+H]^+^	C_21_H_38_O_4_	354.27686	355.28418	1-Linoleoyl glycerol
17.749	[M+H]^+^	C_20_H_34_O_2_	306.25572	307.26303	*Γ*-Linolenic acid ethyl ester
17.775	[M+H-H_2_O]^+^	C_33_H_52_O_5_	528.3822	529.38953	Pachymic acid
18.518	[M+H]^+^	C_16_H_30_O_2_	254.2246	255.23183	Palmitoleic acid
18.577	[M+H-H_2_O]^+^	C_30_H_48_O_3_	456.36039	457.36777	Ursolic acid
18.601	[M+H]^+^	C_18_H_37_NO_2_	299.28224	300.28952	Palmitoylethanolamide
18.962	[M+H]^+^	C_21_H_40_O_4_	356.29248	357.29977	Monoolein
19.038	[M+H-H_2_O]^+^	C_30_H_46_O_3_	454.34482	455.35299	Dehydrotrametenolic acid
19.74	[M+H]^+^	C_18_H_35_NO	281.27173	282.27899	Oleamide
20.128	[M+H]^+^	C_16_H_33_NO	255.25618	256.26346	Hexadecanamide
20.812	[M+H]^+^	C_20_H_34_O_2_	306.25572	307.26303	Linolenic acid ethyl ester
22.464	[M+H]^+^	C_18_H_37_NO	283.2874	284.29468	Stearamide
23.556	[M+H]^+^	C_22_H_43_NO	337.3344	338.34174	Erucamide

**Table 2:** The compounds identified from GPE by LC-MS-MS in negative model.

RT (min)	Adducts	Formula	Measured value	Molecular weight	Name
0.769	[M-H]^−^	C_17_H_27_N_3_O_17_P_2_	607.08107	606.07379	UDP-n-Acetylglucosamine
0.943	[M-H]^−^	C_6_H_12_O_7_	196.05816	195.05075	Gluconic acid
0.945	[M-H]^−^	C_12_H_22_O_11_	342.11583	341.10855	*α*,*α*-Trehalose
0.946	[M-H]^−^	C_6_H_12_O_6_	180.06327	179.05606	L-Sorbose
0.999	[M-H]^−^	C_4_H_6_O_5_	134.02147	133.01421	DL-Malic acid
1.113	[M-H]^−^	C_6_H_8_O_7_	192.02686	191.01958	Citric acid
1.154	[M-H]^−^	C_4_H_6_O_4_	118.02666	117.01942	Succinic acid
1.358	[M-H]^−^	C_7_H_6_O_5_	170.02148	169.01421	Gallic acid
1.87	[2M-H]^−^	C_16_H_24_O_11_	392.13228	391.12427	Shanzhiside
2.756	[M-H]^−^	C_16_H_18_O_9_	354.0948	353.08759	Neochlorogenic acid
3.056	[M-H]^−^	C_16_H_24_O_10_	376.13649	375.12929	Mussaenosidic acid
4.441	[M-H]^−^	C_16_H_24_O_10_	376.13663	375.12943	Loganic acid
4.454	[M-H]^−^	C_16_H_18_O_9_	354.0948	353.08762	Cryptochlorogenic acid
4.529	[M-H]^−^	C_9_H_6_O_4_	178.02656	177.01924	Esculetin
4.554	[M+FA-H]^−^	C_23_H_34_O_15_	550.18934	549.18079	Genipin 1-*O*-*β*-D-gentiobioside
4.663	[M-H]^−^	C_9_H_8_O_4_	180.04225	179.03493	Caffeic acid
4.902	[M-H]^−^	C_16_H_18_O_9_	354.0948	353.08762	Chlorogenic acid
5.028	[M+FA-H]^−^	C_17_H_24_O_10_	388.13649	387.12878	Geniposide
5.69	[M-H]^−^	C_7_H_8_O_2_	124.05246	123.04516	4-Hydroxybenzyl alcohol
5.734	[M-H]^−^	C_9_H_8_O_3_	164.04736	163.04008	3-Coumaric acid
5.847	[M-H]^−^	C_8_H_14_O_4_	174.08906	173.08179	Suberic acid
6.356	[M-H]^−^	C_11_H_12_O_5_	224.06829	223.06102	Sinapic acid
6.384	[M-H]^−^	C_27_H_30_O_16_	610.15473	609.14746	Rutin
6.556	[M-H]^−^	C_21_H_20_O_12_	464.09505	643.08768	Isoquercitrin
6.729	[M-H]^−^	C_25_H_24_O_12_	516.12591	515.11871	Isochlorogenic acid B
6.869	[M-H]^−^	C_27_H_30_O_15_	594.1583	593.15118	Kaempferol-3-*O*-rutinoside
6.884	[M-H]^−^	C_25_H_24_O_12_	516.12587	515.11884	3,5-Dicaffeoylquinic acid
7.057	[M-H]^−^	C_21_H_20_O_11_	448.10017	447.0929	Astragalin
7.234	[M+cl]^−^	C_25_H_24_O_12_	516.12591	551.0954	4,5-Dicaffeoylquinic
8.104	[M-H]^−^	C_15_H_20_O_4_	264.13576	263.12848	(±)-Abscisic acid
8.451	[M-H]^−^	C_15_H_10_O_7_	302.0424	301.03513	Morin
8.516	[M-H]^−^	C_15_H_10_O_6_	286.04741	285.04013	Luteolin
10.273	[M-H]^−^	C_12_H_14_O_4_	222.0889	221.08162	Monobutyl phthalate
11.813	[M-H]^−^	C_16_H_12_O_5_	284.06801	283.06064	Acacetin
12.754	[M-H]^−^	C_18_H_34_O_4_	314.24519	313.23782	(+/-)12(13)-Dihome
15.249	[M-H]^−^	C_31_H_46_O_5_	498.33362	497.32639	Poricoic acid A
17.272	[M-H]^−^	C_32_H_50_O_5_	514.36476	513.35754	3-O-Acetyl-1*α*-hydroxytrametenolic acid, pachymic acid
17.806	[M-H]^−^	C_33_H_52_O_5_	528.38028	527.37286	Oleic acid alkyne
17.917	[M-H]^−^	C_18_H_30_O_2_	278.2242	277.21689	Ursolic acid
18.595	[M-H]^−^	C_30_H_48_O_3_	456.3596	455.35211	Ursolic acid

**Table 3:** Effective ingredients in GPE.

MOL ID	Compound name	Source	Target no.
MOL000273	16*α*-Hydroxydehydrotrametenolic acid	*P. cocos*	2
MOL000275	Trametenolic acid	*P. cocos*	1
MOL000276	7,9(11)-Dehydropachymic acid	*P. cocos*	0
MOL000279	Cerevisterol	*P. cocos*	1
MOL000280	(2*R*)-2-[(3*S*,5*R*,10*S*,13*R*,14*R*,16*R*,17*R*)-3,16-Dihydroxy-4,4,10,13,14-pentamethyl-2,3,5,6,12,15,16,17-octahydro-1H-cyclopenta[a]phenanthren-17-yl]-5-isopropyl-hex-5-enoic acid	*P. cocos*	0
MOL000282	Ergosta-7,22E-dien-3beta-ol	*P. cocos*	1
MOL000283	Ergosterol peroxide	*P. cocos*	1
MOL000285	(2*R*)-2-[(5*R*,10*S*,13*R*,14*R*,16*R*,17*R*)-16-Hydroxy-3-keto-4,4,10,13,14-pentamethyl-1,2,5,6,12,15,16,17-octahydrocyclopenta[a]phenanthren-17-yl]-5-isopropyl-hex-5-enoic acid	*P. cocos*	0
MOL000287	Eburicoic acid	*P. cocos*	0
MOL000289	Poricoic acid	*P. cocos*	0
MOL000290	Poricoic acid A	*P. cocos*	0
MOL000291	Poricoic acid B	*P. cocos*	0
MOL000292	Poricoic acid C	*P. cocos*	0
MOL000296	Hederagenin	*P. cocos*	23
MOL000300	Dehydroeburicoic acid	*P. cocos*	0
MOL001406	Crocetin	*G. jasminoides*	14
MOL001663	3-Epioleanolic acid	*G. jasminoides*	0
MOL001941	Ammidin	*G. jasminoides*	8
MOL004561	Sudan III	*G. jasminoides*	13
MOL000098	Quercetin	*G. jasminoides*	153
MOL000358	Beta-sitosterol	*G. jasminoides*	37
MOL000422	Kaempferol	*G. jasminoides*	72
MOL000449	Stigmasterol	*G. jasminoides*	34
MOL001494	Mandenol	*G. jasminoides*	3
MOL001506	Supraene	*G. jasminoides*	0
MOL001942	Isoimperatorin	*G. jasminoides*	1
MOL002883	Ethyl oleate (NF)	*G. jasminoides*	1
MOL003095	5-Hydroxy-7-methoxy-2-(3,4,5-trimethoxyphenyl)chromone	*G. jasminoides*	26
MOL007245	3-Methylkempferol	*G. jasminoides*	11
MOL009038	GBGB	*G. jasminoides*	0

**Table 4:** Binding energy of effective ingredients in GPE with potential HUA targets.

Compounds	Binding energy (kcal/mol)			
	AKT1	VEGFA	MAPK1	IL-6
Quercetin	-8.1	-6.9	-9.1	-9.4
Kaempferol	-8.1	-5.5	-7.5	-5.9
Hederagenin	-6.5	-7		

**Table 5:** Effect of GPE on plasma uric acid levels in hyperuricemic rats (*μ*g/mL, $\bar{\text{x}}\pm \text{s}$) (*n* = 6).

Groups	UA
BC	3.37 ± 0.14
MC	5.56 ± 0.41^∗∗∗^
PC	3.69 ± 0.06^###^
GPE-HD	4.64 ± 0.70^#^
GPE-MD	4.83 ± 0.25^#^
GPE-LD	3.70 ± 0.28^###^

Note: compared with BC, ^∗^*P* < 0.05, ^∗∗^*P* < 0.01, ^∗∗∗^*P* < 0.001; compared with MC, #*P* < 0.05, ^##^*P* < 0.01, *^###^P* < 0.001.

## Data Availability

The [data type] data used to support the findings of this study are included within the article.
